# Effect of GBF Process Conditions on the Microstructural Characteristic, Melt Quality and Mechanical Properties of Al-Si Alloys with Scrap Addition

**DOI:** 10.3390/ma18050943

**Published:** 2025-02-21

**Authors:** Minji Kim, Kyung Il Kim, Jeong-Keun Lee, Soong-Keun Hyun, Kyung-Taek Kim

**Affiliations:** 1 Research Institute of Intelligent Manufacturing & Materials Technology, Korea Institute of Industrial Technology, Incheon 21999, Republic of Korea; kimminji722@gmail.com (M.K.); kandrew@kitech.re.kr (K.I.K.); 2 Manufacturing Innovation School, Inha University, Incheon 22212, Republic of Korea; skhyun@inha.ac.kr; 3 DONG YANG PISTON Co., Ltd., Ansan 15420, Republic of Korea; jklee@dypiston.co.kr

**Keywords:** Al alloy, Al scrap, gas bubbling filtration, Prefil test, mechanical properties

## Abstract

In this study, the applicability of an Al-Si alloy with 30 wt% added scrap for automobile pistons was evaluated by investigating the melt quality, microstructural characteristics, and tensile properties under modified GBF (gas bubbling filtration) process conditions, including increasing rotor rotation speed and adjusting the air-line supply and the inclination angle of the impeller blade. The melt quality was dramatically improved under modified GBF process conditions, resulting in a very clean melt, with the D.I. value decreasing by 28%, the length of the oxide layer per kilogram decreasing by 65%, and inclusion content decreasing by 97% compared to that of the conventional GBF process conditions. Additionally, the size of primary Si decreased from 40 µm to 27 µm, and the eutectic Si and intermetallic compounds were refined, showing a very fine microstructure. The identified phases included Al_4_Cu_2_Mg_8_Si_7_, Al_x_Cu_y_Ni_z_, and MgO. The ultimate tensile strength was 275 MPa, and the elongation was 6.0%, indicating improved tensile properties compared to those of the conventional GBF process conditions. The fracture behavior changed from a brittle microcleavage fracture mode to a ductile dimple fracture mode as the primary Si, eutectic Si particles, and intermetallic compounds were refined under modified GBF process conditions. These results confirmed that Al-Si alloy with added scrap can be used as a material for automobile pistons.

## 1. Introduction

Recently, with the increasing global greenhouse gas emissions, the average temperature on Earth has increased. Therefore, it is important to achieve carbon neutrality by 2050 [1]. With the increasing importance of carbon neutrality, the environmental issues caused by automotive emissions and energy resource depletion must be addressed urgently. Over the past decades, the automotive industry has seen significant demand for lightweight materials such as aluminum alloys [2]. In particular, the global market for aluminum components is projected to grow at a compound annual rate of approximately 8.3% by 2026, and the global market for Al–Si alloys is expected to grow at an average rate of 2.84% from 2022 to 2030 [3].

Aluminum alloys are widely used in various industries, such as the automotive, aerospace, packaging, construction, cable, and electronics sectors, and their usage is steadily increasing [4]. In particular, automobile makers are increasingly requesting the use of scrap alloys in combustion engine piston alloys while ensuring a reliability of 95% strength in order to achieve carbon neutrality by 2050.

Furthermore, aluminum scrap recycling is considered a crucial factor in advancing toward a sustainable, low-carbon economy. For example, recycling aluminum alloys can reduce the carbon footprint of the production process. Shankar et al. studied methods to effectively sort casting and forging alloys from automotive scrap and proposed quality control techniques [5]. Additionally, research on developing new multi-component alloys by directly melting scrap is actively underway [6]. These processes are environmentally advantageous and require the development of technologies for impurity removal and melt quality control. Recycling aluminum scrap is recognized as an effective approach to mitigating resource depletion while reducing energy consumption by up to 95% and significantly lowering carbon emissions [7]. Moreover, recent research on aluminum scrap alloy casting aims to move beyond conventional downcycling and focus on the development of high-value alloys. This requires precise alloy design and melt quality control technologies [6]. In particular, studies are being conducted to optimize casting performance by managing impurity levels through multi-element alloy design.

To recycle aluminum alloys, it is essential to develop technologies for removing oxides and impurities from scrap, controlling alloy composition, and ensuring the quality control of the melt. The oxides, inclusions, and hydrogen gas present in the aluminum melt contribute significantly to casting defects. Furthermore, when aluminum combines with oxygen in the atmosphere during melting, dross, an impurity, forms on the surface of the melt, leading to quality deterioration [8]. Therefore, melt quality control technologies that employ melt treatment processes before casting must be developed. One such process is gas bubbling filtration (GBF), which is widely used for melt treatment. The GBF process aims to reduce porosity and minimize dross within the melt by optimizing process conditions, such as impeller rotation speed, Ar gas flow rate, and impeller shape [9]. When an impeller is introduced into the melt, it removes hydrogen gas. Additionally, optimizing the impeller shape has been reported to induce different flow patterns within the melt, thereby improving degassing efficiency [10]. Experimental results obtained under optimized GBF process conditions have shown that the maximum tensile strength and elongation of aluminum alloys increase by approximately 30 MPa and 1.5%, respectively, compared with those of alloys produced without GBF treatment [11]. This improvement can be attributed to the reduction of microstructural coarsening and micro-porosity owing to the optimized process conditions. However, while research on aluminum refining is actively progressing, studies on aluminum scrap recycling using GBF processes remain inadequate.

In the present study, the applicability of scrap-added Al-Si alloy for automobile pistons was evaluated by investigating the melt quality, microstructural characteristics, and tensile properties under various GBF process conditions, including increasing rotation speed and adjusting the air-line supply and inclination angle of the impeller blade. The main GBF process conditions, such as impeller shape, rotor rotation speed, and Ar gas flow rate, were verified through a water model test, and the melt quality, microstructural characteristics, and tensile properties at room temperature were evaluated based on variations in rotor rotation speed and impeller shape. In addition, fracture behavior was investigated through fracture surface and cross-sectional analysis of the tensile test specimen.

## 2. Materials and Methods

### 2.1. Evaluation of Water Modeling

**Figure 1:**
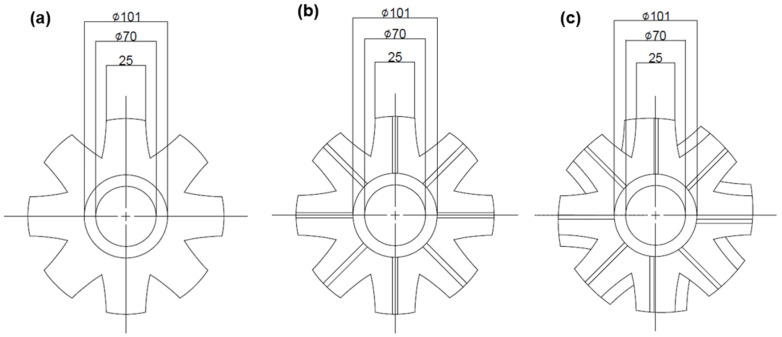
Design of impeller for GBF process: inclination angle [12]: (**a**,**b**) 0°, (**c**) 30°; air-line: (**a**) 0, (**b**,**c**) 8.

Through a water model test with a viscosity similar to that of Al melt, the GBF process conditions were selected by verifying the effect of rotor rotation speed, Ar gas flow rate, and impeller blade shape on the vortex formation in the melt, as well as the size and distribution of Ar gas bubbles. The crucible used in the water model test was manufactured using transparent acrylic and had the same shape and size as that used in the Al piston manufacturing process. Likewise, the rotor and impeller were manufactured using graphite, as shown in Figure 1 and Table 1 The water model test conditions included a rotor rotation speed of 300 to 500 RPM, an Ar gas flow rate of 20 L/min and 40 L/min, and an impeller shape as shown in Figure 1 and Table 1. Table 2 shows the GBF process conditions selected through the water model test.

### 2.2. Material and Casting

The raw materials used in this study were a primary Al-12%Si alloy ingot for Al pistons and a scrap ingot made by re-melting chip-shaped scrap produced during piston machining. A total of 30 wt% of the scrap ingot was added to the primary ingot. The chemical composition of the raw materials consisted of Al-12%Si alloy with Fe, Cu, Mn, Ni, and Mg additions, as shown in Table 3, and the chemical composition of the primary and scrap ingots was identical.

The casting was produced by melting the graphite crucible furnace with a 500 kg capacity at approximately 720 ± 10 °C, followed by a melt purification process. The melt purification process was conducted in three stages using the GBF equipment (GBF machine-500, DONGWOOROTECH, Changnyeong-gun, Republic of Korea). First, flux was added to remove oxides and inclusions, followed by the addition of powdered phosphorus to refine the primary Si phase. Finally, Ar gas was injected at a rate of 40 L/min using an impeller for degassing.

The impeller used for the degassing process was made of graphite to facilitate operation in the melt and had eight blades, as shown in Figure 1. Figure 1a illustrates the impeller used in the conventional GBF process, while Figure 1b shows the same impeller shape with Ar gas flow holes of 10 mm width and depth added at the bottom of each blade. Figure 1c shows the impeller shape from Figure 1b with an additional 30° inclination angle [12]. Table 2 presents the GBF process conditions used in this study.

Following the melt purification process, the melt was injected into a tool steel mold (SKD61) at approximately 700 ± 10 °C to produce specimens. The heat treatment process for the castings involved a solution treatment at 480 °C in air for 2 h, followed by water quenching and artificial aging treatment at 230 °C for 5 h (T5 treatment). The specimens produced under each process condition are listed in Table 3.

### 2.3. Evaluation of Melt Quality and Microstructural Characteristics

Internal defects were evaluated using D.I. values and Prefil tests (Prefil–Footprinter, ABB, Saint-Laurent, QC, Canada). The D.I. value is required to be 1.0 or lower, and the smaller the density index, the lower the tendency for porosity formation. The D.I. value was measured using vacuum solidification equipment, and the density values of specimens solidified under atmospheric and vacuum conditions were obtained using Equation (1):

(1) $$\rho =\frac{W_{1}}{W_{1}-W_{2}}$$

where ρ represents the measured density (g/cm^3^), *W*_1_ is the specimen’s weight in air (g), and *W*_2_ is the specimen’s weight in water (g). The D.I. value was calculated using the values obtained from Equation (1) and applying the formula in Equation (2):

(2) $$\frac{A-B}{A}\times 100$$

where *A* is the density of the specimen solidified under atmospheric conditions, and B is the density of the specimen solidified under vacuum conditions [13].

For the Prefil tests, the melt flow time and weight changes through a ceramic filter were analyzed after GBF treatment of the Al piston alloy, and the oxides and inclusions accumulated on the filter were examined microscopically to quantitatively evaluate the molten metal quality. The D.I. value was measured 80 times, and the Prefil test was conducted 10 times. The mean values were calculated after excluding the maximum and minimum values.

A microstructural analysis was conducted using scanning electron microscopy (SEM, JSM–5510, JEOL, Tokyo, Japan) to examine the refinement of the Si phases and the presence of intermetallic compounds. The size of the primary Si phase was measured using optical microscopy (OM), with measurements taken 50 times. The mean value was calculated after excluding the maximum and minimum values.

Energy–dispersive X-ray spectroscopy (EDS) was used to compare the phases and fractions of the microstructures. Through X-ray diffraction (XRD, SmartLab, Rigaku, Tokyo, Japan), the phases were analyzed under various GBF process conditions. The measurement conditions for each material were set with an X-ray diffraction angle (2θ) ranging from 20° to 80°, and changes in diffraction peaks were observed at a scanning speed of 4°/min. Additionally, an incident slit size of 2 mm was used, and Cu Kα radiation (λ = 1.5406 Å) was applied.

### 2.4. Evaluation of Tensile Properties

The tensile properties of the alloys were evaluated using a tensile testing machine (MR–J2S–200A, MITSUBISHI, Tokyo, Japan) at room temperature in accordance with the ASTM E8M standard [14]. The tensile specimens were machined to a gauge length of 30 mm and a diameter of 6 mm and were tested at a strain rate of 1.5 mm/min. The ultimate tensile strength (UTS) and elongation values were measured using 10 specimens, and the mean values were calculated after excluding the maximum and minimum values.

For the specimens that fractured during tensile testing, the microstructures of the surface and cross sections were observed under different conditions to analyze the fracture behavior of the alloys. Additionally, phase analysis and phase fraction comparisons of the microstructures were conducted using EDS.

## 3. Results and Discussion

### 3.1. Water Model Test

**Figure 2:**
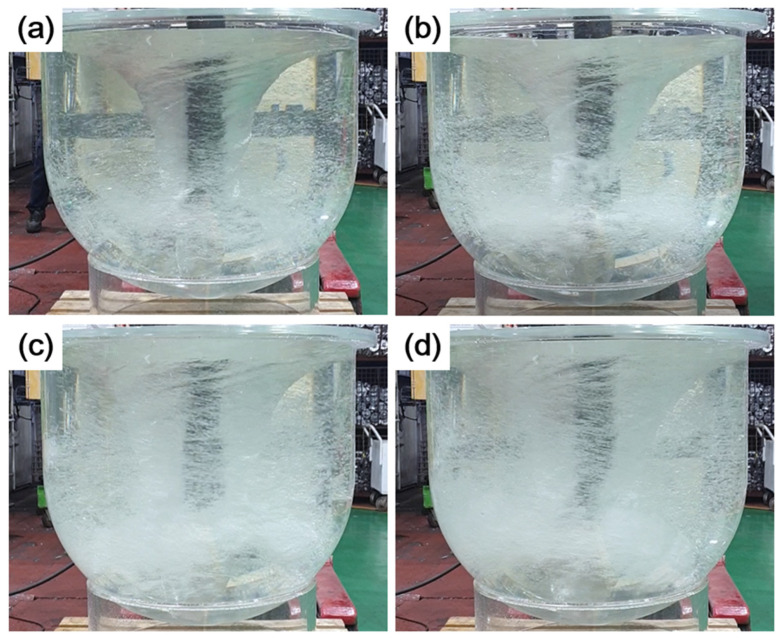
Water modeling results based on RPM and Ar gas flow rate (L/min): (**a**) 300-20, (**b**) 300-40, (**c**) 550-20, (**d**) 550-40.

Figure 2 shows the water model test results illustrating the effects of changes in rotor rotation speed and Ar gas flow rate under conventional GBF process conditions (condition #1). As the rotor rotation speed increased from 300 RPM to 550 RPM, the vortex structure expanded, and a large number of bubbles formed. Additionally, increasing the Ar gas flow rate from 20 L/min to 40 L/min at the same rotor rotation speed resulted in a larger number of fine bubbles that were generated and evenly distributed. However, air pockets and a dead zone appeared in the longitudinal direction of the rotor and at the bottom of the crucible, respectively.

Based on the results of these water model tests, the Ar gas flow rate was set to 40 L/min, and a water model test was conducted according to changes in the rotor rotation speed and impeller blade shape.

**Figure 3:**
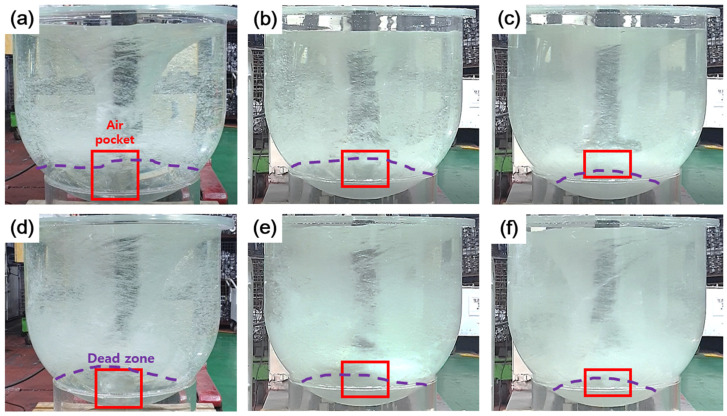
Air pocket (Red box) and dead zone (Purple dotted line) distribution in impeller shape optimization via water modeling: (**a**) 300-40 alloy #1, (**b**) 300-40 alloy #2, (**c**) 300-40 alloy #3, (**d**) 550-40 alloy#1, (**e**) 550-40 alloy #2, (**f**) 550-40 alloy #3.

Figure 3 shows the water model test results based on variations in rotor rotation speed and impeller blade shape under the condition where the Ar gas flow rate was set to 40 L/min. Figure 3a,b,d,e show the results of water model tests with increasing rotor rotation speed and the addition of air-lines to the impeller blades. It was observed that when an air-line was added to the impeller blade (condition #2), the air pocket and dead zone were reduced, and the main stream of Ar gas shifted downward toward the bottom of the crucible.

As observed in Figure 3b,c,e,f, adding an inclination angle to the impeller blade (condition #3) further reduced the air pockets and dead zones. Additionally, the main stream of Ar gas was directed further downward. The size and distribution of Ar gas bubbles became finer and more uniform by adding air-lines and an inclination angle to the impeller blade.

Therefore, applying both an inclination angle and an air-line to the impeller is expected to improve the degassing effect due to the main stream of Ar gas being generated at the bottom of the crucible and the formation of finer bubbles. In other words, the formation of fine bubbles at the bottom of the crucible increases the surface area of the bubbles, slows their rise, and allows them to stay in the melt longer, improving degassing efficiency [15]. However, in the case of the inclination angle, it has been reported that when an inclination angle greater than 30° is applied, the blades become closer to each other, reducing the space between them and decreasing stirring efficiency [12].

Based on the results of the above water model tests, the rotor rotation speed and Ar gas flow rate were fixed at 550 RPM and 40 L/min, respectively, under the GBF process conditions, and the melt quality, microstructural characteristics, and tensile properties were investigated according to changes in the shape of the impeller blade, such as the application of an air-line and adjustment of the inclination angle of the impeller blade.

### 3.2. Melt Quality

**Figure 4:**
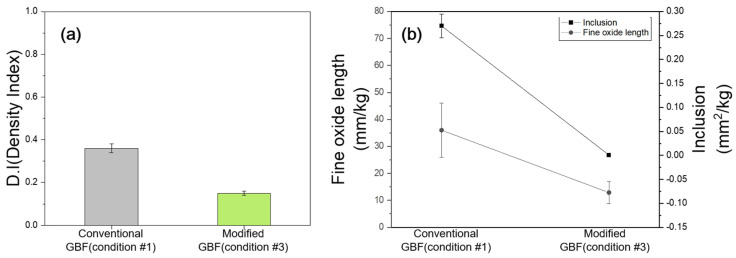
D.I. values and Prefil test results for melt quality: (**a**) D.I. value, (**b**) fine oxide length and inclusion.

The D.I. values under the conventional GBF conditions (GBF condition #1) and modified GBF conditions (GBF condition #3) are shown in Figure 4a. The automobile manufacturer’s requirement for the D.I. value is less than 1.0, which was already satisfied at 0.36 under the conventional GBF process conditions. However, under the modified GBF process conditions, it further decreased by approximately 28%, reaching 0.26. Gas voids typically exhibit smooth and rounded shapes and can be reduced by removing hydrogen gas present in the melt [16]. Additionally, if oxides and inclusions are not removed from the melt during casting, these impurities can act as nucleation sites, causing gas to accumulate in the vicinity and leading to voids [17].

Therefore, this result indicates that residual hydrogen gas, oxides, and inclusions in the melt were more effectively eliminated using the modified GBF process conditions.

Figure 4b shows the results of oxide and inclusion measurements obtained through the Prefil tests conducted on the melt. We measured the sizes of the oxides and inclusions accumulated on the Prefil ceramic filter and calculated the weight of the input melt and the filter size to perform a quantitative evaluation. The mean oxide length under the conventional GBF process conditions (GBF condition #1) was 36 mm/kg, whereas under the modified GBF process conditions (GBF condition #3), it decreased to 12.9 mm/kg, representing a reduction of approximately 64%.

For inclusions, under the conventional GBF process conditions (GBF condition #1), the mean area covered per mass of the melt was 0.027 mm^2^/kg, whereas under the modified GBF process conditions (GBF condition #3), this value decreased by 97% to 0.0007 mm^2^/kg. These results confirmed that the modified GBF process conditions (GBF condition #3) led to a significant reduction in the oxides and inclusions present in the melt.

Additionally, both the oxide length and the inclusion area showed significant reductions in standard deviation when transitioning from the conventional GBF treatment to the modified GBF treatment, indicating an overall improvement in process consistency. Consequently, these results indicate that an extremely clean melt can be secured by optimizing GBF process conditions.

### 3.3. Microstructural Characteristics and XRD Analysis

**Figure 5:**
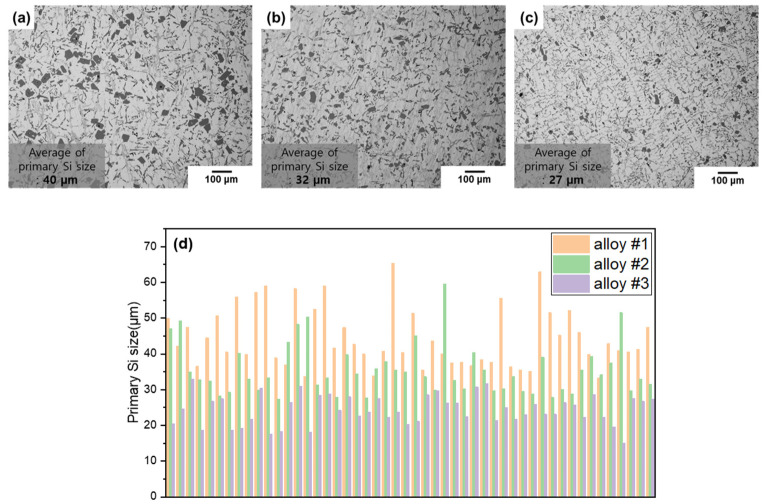
Optical micrographs and the size of primary Si in the Al-12%Si alloy under different GBF conditions: (**a**) alloy #1, (**b**) alloy #2, (**c**) alloy #3. (**d**) Graph showing the size of primary Si under different GBF conditions.

The optical micrographs of each alloy according to changes in GBF process conditions are shown in Figure 5. As shown in Figure 5, when an air-line and inclination angle were applied to the impeller blade, the mean size of the primary Si decreased from 40 µm (Alloy #1) to 27 µm (Alloy #3). Additionally, the eutectic Si and intermetallic compounds became finer.

In general, coarse primary Si, eutectic Si, and intermetallic compounds in the Al matrix act as stress concentration points, which cause a decrease in tensile properties. Therefore, an improvement in tensile properties is expected through a refined microstructure [11].

**Figure 6:**
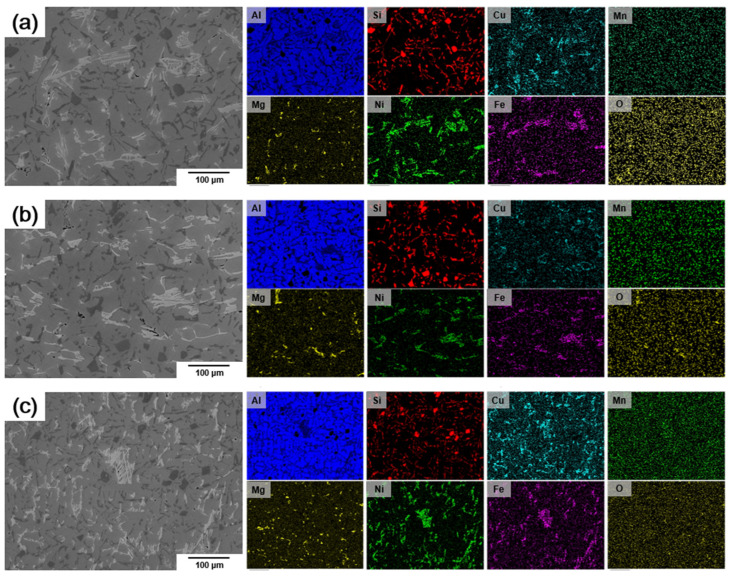
Backscatter electron (BSE) images and energy–dispersive X-ray spectroscopy (EDS) analysis of Al–12%Si alloys: (**a**) alloy #1, (**b**) alloy #2, (**c**) alloy #3.

Figure 6 shows the elemental mapping images of each alloy, demonstrating that Si, Mg, Ni, and Cu elements became finer and were uniformly distributed when air-lines and an inclination angle were applied to the impeller blade. In particular, a significant reduction in oxygen content was observed. Additionally, Ni, Cu, and Fe elements were detected at the same location.

**Figure 7:**
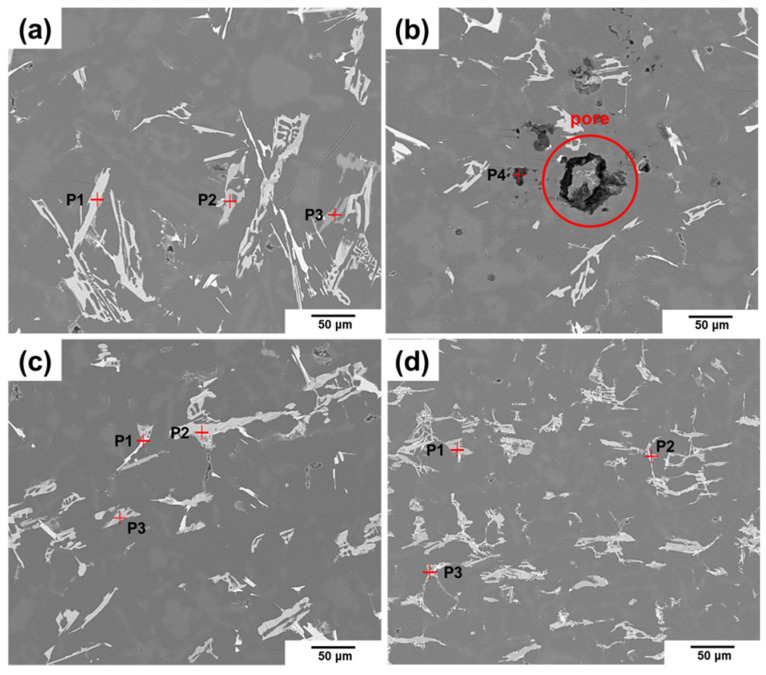
Component analysis of Al–12%Si alloys was conducted using scanning electron microscopy (SEM) along with energy–dispersive X-ray spectroscopy (EDS) for detailed element identification: (**a**,**b**) alloy #1, (**c**) alloy #2, (**d**) alloy #3.

The SEM images and EDS analysis results of each alloy are shown in Figure 7 and Table 4, respectively. In Alloy #1, needle-like compounds were found, as shown in Figure 7a,b. In particular, in the black-colored compounds with a particle-like shape, Al and Mg elements were detected along with O and C elements.

Additionally, pores approximately 50 µm in size were observed around the inclusions. In contrast, the microstructures of Alloys #2 and #3 show a fine microstructure without micropores, as shown in Figure 7c,d, and O and C elements were not detected.

These results show that Alloy #3, with both an air-line and an inclination angle applied to the impeller blade, exhibits a sound microstructure free of micropores, which verifies the D.I. value and Prefil test results.

**Figure 8:**
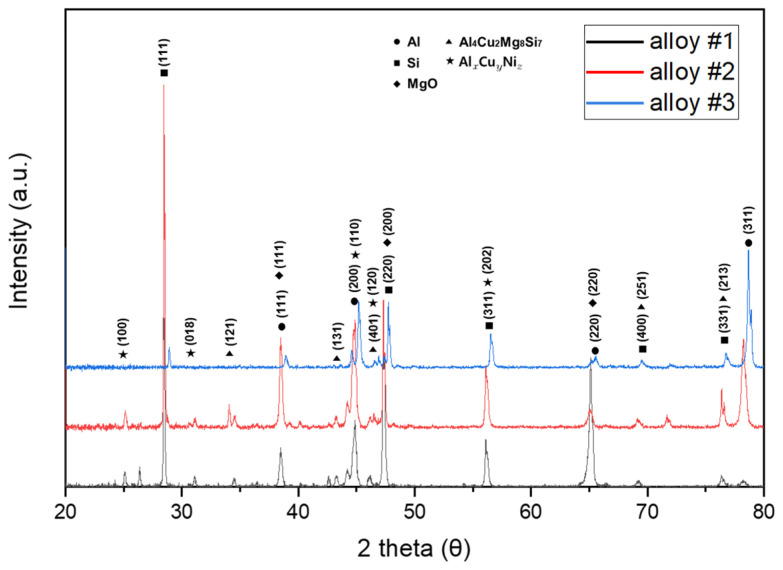
X-ray diffraction analysis result under GBF conditions.

Figure 8 shows the XRD analysis results based on changes in GBF process conditions, focusing on the intermetallic compound phase. Although the GBF process conditions varied, the chemical composition of the alloy remained the same, and the intermetallic compounds identified through XRD were identical, including Al_4_Cu_2_Mg_8_Si_7_, the Al_x_Cu_y_Ni_z_ phase, and MgO.

### 3.4. Tensile Properties

**Figure 9:**
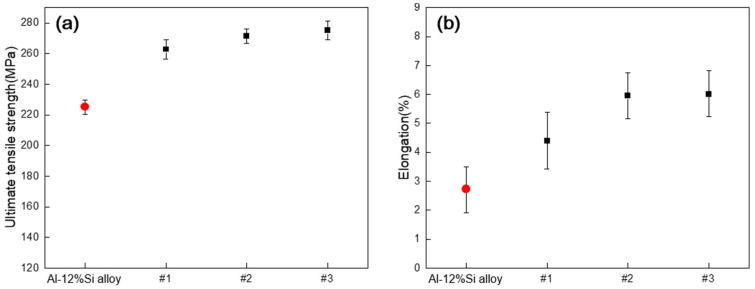
Tensile properties of Al–12%Si alloys at room temperature: (**a**) Ultimate tensile strength, (**b**) Elongation (Al–12%Si alloy [18], #1, #2, #3).

Figure 9 shows the ultimate tensile strength and elongation at room temperature under varying GBF process conditions. Generally, primary Al–12%Si alloy has an ultimate tensile strength of approximately 220 MPa and an elongation of approximately 2.7% [18].

For Alloy #1, the conventional GBF process was conducted, resulting in an ultimate tensile strength of approximately 260 MPa and an elongation of approximately 4.5%, indicating improved tensile properties compared with those of the primary Al–12%Si alloy.

For Alloy #2, using the modified GBF process (condition #2), an ultimate tensile strength of approximately 270 MPa and an elongation of approximately 5.9% were achieved.

For Alloy #3, using the modified GBF process (condition #3), an ultimate tensile strength of approximately 275 MPa and an elongation of approximately 6% were obtained. The elongation of Alloy #3 was almost the same as that of Alloy #2; however, the tensile strength increased by 5 MPa.

Therefore, from Alloy #1 to Alloy #3, the ultimate tensile strength increased by approximately 6%, whereas the elongation increased by approximately 35%.

The elongation of the Al–12%Si alloy was influenced by the size and morphology of the primary Si phases and intermetallic compounds, as well as the amount of oxides. The improvement in the GBF process conditions and the application of an inclined impeller blade with increased agitation and degassing efficiency were expected to refine the constituent phases and reduce the oxide content during solidification, resulting in increased elongation.

This result is consistent with the previously described oxide mapping results, where the amount of oxide decreased from Alloy #1 to Alloy #3.

**Figure 10:**
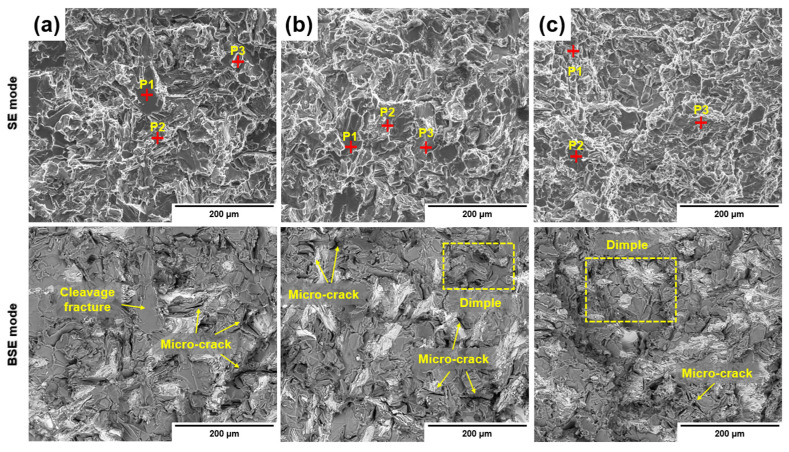
EDS analysis results for fracture surfaces of Al–12%Si alloys were obtained using SEM images to provide detailed insights into the element distribution on the fracture surfaces: (**a**) #1 alloy, (**b**) alloy #2, (**c**) alloy #3.

The fracture behavior of the alloy under different GBF process conditions and the fractured surfaces of the tensile specimens were observed using SEM. Figure 10 shows the fractographs of the tensile test samples, and Table 5 provides a detailed tabulation of the EDS results for the fractured surfaces.

Microcracks measuring 10–100 µm in size were observed in Alloy #1, and these microcracks were generated by unrefined needle-like eutectic Si during crack propagation initiated in coarse primary Si. Moreover, when large cracks formed and reached the Al matrix during crack propagation, the propagation temporarily stopped. As the load increased, the crack changed its direction, and new cracks appeared due to the fractured eutectic Si [18].

The fracture surface analysis results showed irregular cleavage fractures in areas containing the Si phases and intermetallic compounds. Furthermore, the results of EDS analysis of the fractured surface (Figure 10a) revealed brittle fractures in the Al_x_Cu_y_Ni_z_ and eutectic Si phases. According to previous studies, when observing the microstructure, primary Si, eutectic Si phases, and intermetallic compounds appear to exist independently of the Al matrix. However, after etching and analyzing the fractured surface, the Si phases and intermetallic compounds were found to be interconnected [11].

Therefore, when cracks formed, they propagated along the Al–Si interface, acting as stress concentrators and causing the fracture of intermetallic compounds within the alloy [11].

For Alloy #2 (Figure 10b), microcracks were also observed as in Alloy #1; however, their sizes were smaller than those in Alloy #1. Additionally, a transition from brittle microcleavage fracture to ductile dimple fracture was observed.

For Alloy #3 (Figure 10c), almost no microcracks were observed, and ductile fractures were predominantly observed over a wide area.

According to the EDS analysis (Table 4), the fractured surfaces of Alloy #2 and Alloy #3 exhibited significantly less oxidation than those of Alloy #1. Furthermore, the increase in elongation observed through tensile testing was attributed to the ductile fracture behavior of Alloy #2 and Alloy #3, along with the reduced presence of oxides. Additionally, while the Al_x_Cu_y_Ni_z_ phase in Alloy #1 exhibited microcleavage fractures approximately10 µm in size, in Alloy #2 and Alloy #3, the size of the fractured areas decreased, and ductile fractures occurred primarily in the eutectic Si phase. These results indicate that as the GBF process conditions were optimized, the efficiency of the GBF treatment increased from Alloy #1 to Alloy #3, leading to a transition in the fracture behavior of the Si phases and intermetallic compounds from brittle to ductile. Microcleavage fractures were observed in small regions, whereas ductile fractures were predominant over wider areas.

**Figure 11:**
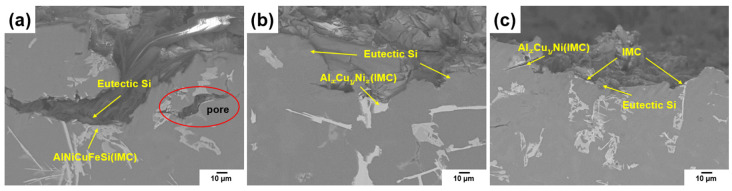
SEM images of longitudinal sections of Al–12%Si alloys under different GBF process conditions: (**a**) alloy #1, (**b**) alloy #2, (**c**) alloy #3.

Figure 11 shows the results of the fractured cross-sectional surfaces of the tensile specimens. As shown in Figure 11a, in Alloy #1, unrefined intermetallic compounds of the Al_x_Cu_y_Ni_z_ phase, along with eutectic Si phases, were observed beneath the fractured surface. Cracks initiated and propagated along the eutectic Si phases, causing microcracks, and fractures were presumed to have occurred in the intermetallic compound phases along these cracks. Additionally, large pores were observed near the surface.

For Alloy #2 (Figure 11b), intermetallic compounds of the Al_x_Cu_y_Ni_z_ phase and eutectic Si phases were detected beneath the fractured surface. Similarly, under condition #3 (Figure 11c), intermetallic compounds and eutectic Si phases were observed beneath the fractured surface.

In particular, in the vicinity where sharp cracks initiated, intermetallic compounds of the Al_x_Cu_y_Ni_z_ phase were detected, and cracks were presumed to have formed due to the presence of needle-like eutectic Si phases during fracture.

## 4. Conclusions

In this study, the melt quality, microstructural characteristics, and tensile properties of Al-Si alloys for automobile pistons with 30 wt% added scrap, having the same chemical composition, were evaluated according to changes in GBF process conditions, and the following conclusions were obtained.

(1) As a result of verifying the flow phenomenon of the melt and the size and distribution of Ar gas bubbles through a water model test, the most stable flow behavior of the melt was observed at a rotor rotation speed of 550 RPM, an Ar gas flow rate of 40 L/min, and the application of an air-line and an inclination angle to the impeller blade.
(2) As a result of the melt quality evaluation, as the rotor rotation speed increased and an air-line and inclination angle were applied to the impeller blade, the D.I. value decreased by 28%, from an average of 0.36 (Alloy #1) to 0.26 (Alloy #3), and the length of the oxide layer per kilogram decreased by 64%, from 36 mm (Alloy #1) to 12.9 mm (Alloy #3). Additionally, the inclusion content decreased by 97%, from 0.027 mm^2^/kg (Alloy #1) to 0.0007 mm^2^/kg (Alloy #3).
(3) The size of primary Si decreased from 40 µm (Alloy #1) to 27 µm (Alloy #3), and the eutectic Si and intermetallic compounds were refined as the rotor rotation speed increased and an air-line and an inclination angle were applied to the impeller blade. However, the formed phases remained the same as those in the primary alloy, including Al_4_Cu_2_Mg_8_Si_7_, Al_x_Cu_y_Ni_z_, and MgO.
(4) As the rotor rotation speed increased and an air-line was applied to the impeller blade, the ultimate tensile strength increased from 260 MPa (Alloy #1) to 270 MPa (Alloy #2), and the elongation increased from 4.5% (Alloy #1) to 5.9% (Alloy #2). When the impeller with the same air-line and a blade inclination of 30° was applied, the tensile strength increased from 270 MPa (Alloy #2) to 275 MPa (Alloy #3), and the elongation increased from 5.9% (Alloy #2) to 6.0% (Alloy #3).
(5) The fracture behavior changed from a brittle microcleavage fracture mode to a ductile dimple fracture mode due to the refinement of primary Si, eutectic Si particles, and intermetallic compounds under modified GBF process conditions.

## Figures and Tables

**Table 1:** Specimens under different process conditions.

Number	#1	#2	#3
Alloy	Al–12%Si + 30% scrap		
Impeller shape (inclination angle)	0°	0°	30°
Impeller shape (air-line)	none	8 lines	
GBF process	Conventional	Modified	

**Table 2:** Conditions for conventional and modified GBF processes.

No.	Process	Conventional Process		Modified Process		Ar Gas Flow Rate
		Time (s)	RPM	Time (s)	RPM	
1	Flux input	240	360	90	550	40 L/min
2	P addition	600	360	600	450	
3	Degassing	600	320	600	400	
Total time		24 min		22 min		

**Table 3:** Chemical composition (wt%) of the Al–12%Si alloy in this study.

Elements	Si	Fe	Cu	Mn	Mg	Ni	P	Al
Content	12.44	0.41	1.07	0.07	1.12	1.11	0.003	Bal.

**Table 4:** EDS analysis results of Al–12%Si alloys.

Element (wt%)	#1				#2			#3		
	P1	P2	P3	P4	P1	P2	P3	P1	P2	P3
C	–	–	–	10.85	–	–	–	–	–	–
O	–	–	–	36.10	–	–	–	–	–	–
Mg	–	–	24.31	08.10	–	–	–	–	–	–
Al	58.64	70.65	20.23	42.23	48.35	58.68	71.44	69.00	66.11	65.29
Si	05.92	01.79	30.14	02.72	–	05.66	01.95	01.47	–	01.40
Fe	06.82	06.74	–	–	–	07.20	07.19	05.68	–	–
Ni	22.64	20.82	–	–	27.05	18.77	19.43	20.83	12.90	14.55
Cu	05.98	–	15.33	–	24.60	09.69	–	03.02	20.99	18.76

**Table 5:** EDS analysis results for fracture surfaces of Al–12%Si alloys.

Element (wt%)	#1			#2			#3		
	P1	P2	P3	P1	P2	P3	P1	P2	P3
Al	38.46	63.33	67.74	13.86	64.03	44.04	57.73	26.86	60.88
Si	55.21	03.04	32.26	86.14	04.61	55.11	11.52	73.14	01.14
Fe	01.25	08.14	–	–	05.74	–	05.19	–	–
Ni	02.55	25.49	–	–	18.42	–	17.08	–	16.67
Cu	02.52	–	–	–	07.23	00.84	08.48	–	21.31

## Data Availability

The original contributions presented in this study are included in the article. Further inquiries can be directed to the corresponding author.

## References

[1] Saevarsdottir G., Magnusson T., Kvande H. (2021). Reducing the Carbon Footprint: Primary Production of Aluminum and Silicon with Changing Energy Systems. J. Sustain. Metall..

[2] Dash S.S., Chen D. (2023). A Review on Processing–Microstructure–Property Relationships of Al–Si Alloys: Recent Advances in Deformation Behavior. Metals.

[3] Soo V.K., Peeters J., Paraskevas D., Compston P., Doolan M. (2018). Sustainable Aluminum Recycling of End–of–Life Products: A Joining Techniques Perspective. J. Clean. Prod..

[4] Kang N., Coddet P., Liao H., Baur T., Coddet C. (2016). Wear Behavior and Microstructure of Hypereutectic Al–Si Alloys Prepared by Selective Laser Melting. Appl. Surf. Sci..

[5] Shankar S., Söderhjelm C., Apelian D. (2024). Classifcation of Automotive Aluminum Scrap into Cast and Wrought Alloys via Particle Size Analysis. J. Sustain. Metall..

[6] Sanchez J.M., Galarraga H., Marquez I., Cortazar M.G. (2025). High-throughput CALPHAD-guided design and experimental study on the development of a novel multicomponent as-cast Al-Si-Cu-Zn-Fe-Mn-Mg based alloy through the direct melting of post-consumer scrap. J. Alloys Compd..

[7] Kleinhans R., Jugert C., Pintore M., Volk W. (2024). Impact of Scrap Impurities on AlSi7Cu0.5Mg Alloy Flowability Using Established Testing Methods. Recycling.

[8] Fintova S., Kubena I., Trsko L., Hornik V., Kunz L. (2020). Fatigue Behavior of AW7075 Aluminum Alloy in Ultra–High Cycle Fatigue Region. Mater. Sci. Eng. A.

[9] Lee C.D., So T.I., Shin K.S. (2016). Effect of Gas Bubbling Filtration Treatment on Micro Porosity Variation in A356 Aluminum Alloy. Acta Metall..

[10] Saternus M., Merder T. (2018). Physical Modelling of Aluminum Refining Process Conducted in Batch Reactor with Rotary Impeller. Metals.

[11] Zhang G.H., Zhang J.X., Li B.C., Cai W. (2011). Characterization of Tensile Fracture in Heavily Alloyed Al–Si Piston Alloy. Mater. Int..

[12] Yamamoto T., Suzuki A. (2018). Investigation of Impeller Design and Flow Structures in Mechanical Stirring of Molten Aluminum. J. Mater. Process. Technol..

[13] Djurdjević M.B., Odanović Z., Pavlović-Krstić J. (2010). Melt Quality Control at Aluminum Casting Plants. Metall. Mater. Eng..

[14] (2024). Standard Test Methods for Tension Testing of Metallic Materials.

[15] Mancilla E., Cruz-Mendez W., Ramirez-Argaez M., Gonzalez-Rivera C., Ascanio G. (2018). Experimental Measurements of Bubble Size Distributions in a Water Model and Its Influence on the Aluminum Kinetics Degassing. Can. J. Chem. Eng..

[16] Anson J.P., Gruzleski J.E. (1999). The Quantitative Discrimination between Shrinkage and Gas Microporosity in Cast Aluminum Alloys Using Spatial Data Analysis. Mater. Charact..

[17] Jang H.S., Kang H.J., Park J.Y. (2020). Effect of Casting Conditions for Reduced Pressure Test on Melt Quality of Al–Si Alloy. Metals.

[18] Okayasu M., Miyamoto Y., Morinaka K. (2015). Material Properties of Various Cast Aluminum Alloys Made Using a Heated Mold Continuous Casting Technique with and without Ultrasonic Vibration. Metals.

